# Assessment of the Impact of a One Health Approach‐Based Training on Poultry Rearing and Farm Biosecurity Management in Bangladesh

**DOI:** 10.1002/vms3.70843

**Published:** 2026-02-07

**Authors:** Meherjan Islam, Ayona Silva‐Fletcher, Md. Ershadul Haque, Rashed Mahmud, Fiona Tomley, Md. Ahasanul Hoque

**Affiliations:** ^1^ Department of Medicine and Surgery Chattogram Veterinary and Animal Sciences University Chattogram Bangladesh; ^2^ Department of Clinical Science and Services Royal Veterinary College, University of London Hatfield UK; ^3^ Department of Pharmacy International Islamic University Chattogram Chattogram Bangladesh; ^4^ Department of Pathobiology and Population Science Royal Veterinary College, University of London Hatfield UK

**Keywords:** Biosecurity, collaboration, One Health, poultry farming, training, training impact

## Abstract

**Introduction:**

Biosecurity measures are crucial for disease prevention and sustainable poultry farming; however, many farmers in Bangladesh do not have access to training in biosecurity and farm management. This study evaluated whether biosecurity training, delivered within a One Health framework, would lead to farmers adopting improved biosecurity measures in their farming practices.

**Methodology:**

The impact of a 2‐day training programme was evaluated on 88 farms across 12 upazilas in the Chattogram district, with assessments conducted immediately after the training and at least 4 months later. Data collection involved farm visits, using a researcher‐led questionnaire and evaluation through direct observation.

**Results:**

The *t*‐test results showed an absolute increase in mean farm scores from 12.3 to 14.8, representing 9% improvement in farm biosecurity and management practices, irrespective of the farm production system, farmers' education and experience levels. Descriptive analysis indicated that improvement percentage (IP) in operational biosecurity, such as cleaning feeders and drinkers (65%), using separate clothes and shoes (64%) and waste management (58%) were the most improved practices. Dealer‐based Sonali farms showed higher IP for disinfectant use (83%), sick bird isolation (88%) and improved brooding management (46%) (*p* < 0.05). Farmers with the highest education level maintained 2‐week intervals between two batches (IP 33%, *p* = 0.004). Less‐experienced farmers improved shed cleaning processes more (42%) and more‐experienced farmers improved the feed storage system (55%) and vaccine transportation (38%) (*p* < 0.05).

**Conclusion:**

The results indicate that using a One Health approach in training can effectively influence various aspects of farm biosecurity and management practices, leading to positive behavioural changes.

**Summary:**

- Collaborative training using a One Health approach can change biosecurity practices and poultry farmer behaviours, reducing zoonoses and public health.
- Financial constraints are a major hindrance for poultry farmers to adopt proper biosecurity measures; however, training can influence the farmers to some extent.
- Operational biosecurity measures are more adoptable by the farmers, irrespective of farm types.

## Graphical abstract

Graphical presentation of the methodology of the paper entitled “Assessment of the impact of a One Health approach‐based training on poultry rearing and farm biosecurity management in Bangladesh”.

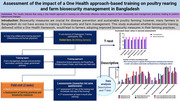

## 1 Introduction

The poultry industry is an important sub‐sector of livestock production in Bangladesh. It contributes 1.81% of the nation's gross domestic product and supplies 22%–27% of animal‐sourced foods (Anon 2025; Howlader et al. 2022). Chicken is the most widely consumed meat, regardless of the religious, economic, social, and demographic differences among people (Rahman et al. 2017). The support of both public and private sectors has led to the poultry industry's transition in recent years from predominantly small‐scale to commercial, medium‐ and large‐scale production systems (Hamid et al. 2017). Of the four main production types, viz. exotic broiler, exotic layer, Sonali (F1 cross of Rhode Island Red males and Egyptian Fayoumi females), and Indigenous deshi chickens, the first three are commercially reared, whilst deshi are reared in semi‐scavenging systems (Hennessey et al. 2021).

Commercial poultry farmers often start farming at small‐ and medium‐scale with little or no technical training, insufficient capital, and poor and inappropriate infrastructure, and many are dependent on feed dealers for credit and advice (Hennessey et al. 2021; Rahman et al. 2021). Sudden outbreaks of disease cause great losses (Al Mamun et al. 2019), and farmers may not understand that lack of biosecurity contributes to outbreaks (Khatun et al. 2019). Thus, they have the potential to benefit financially as implementing farm biosecurity is less costly than disease recovery measures (Khatun et al. 2019).

Appropriate and comprehensive biosecurity measures can significantly reduce or prevent the introduction, transmission, and persistence of pathogens in flocks (Delpont et al. 2023). This protects poultry from serious infectious diseases and reduces risks to humans of zoonotic infections (Leibler et al. 2008). For example, poultry flocks reared with limited biosecurity are more likely to become infected by avian influenza viruses (such as H9N2 and H5N1) and have enhanced potential for zoonotic spillover (Hyder et al. 2023). By reducing disease transmission, biosecurity supports efficient commercial farming, improves food security, and further safeguards public health by reducing the need for antimicrobial use (AMU), thus curbing antimicrobial resistance (AMR) (Collignon and McEwen 2019; FAO 2007). Effective biosecurity practices can also prevent devastating economic and social consequences of disease outbreaks in small family farms in Bangladesh (Islam et al. 2023).

Training programmes can significantly increase farmers' knowledge, awareness, and skills about improved farming practices, leading to better decision‐making and productivity (Rasanjali et al. 2021). While there is evidence that such training leads to knowledge gains about biosecurity practices, the impact on real‐life changes is often limited (Cui et al. 2019; Dione et al. 2020; Panda et al. 2024). Barriers to adoption of improved practices on farms include limited financial resources, reluctance to change due to fear of failure, uncertainty about outcomes of new methods, strong cultural or traditional beliefs, and failure of the training approach to convince farmers that improving biosecurity will reduce disease and improve their livelihoods (Kirui 2014; Rodriguez et al. 2009). Recently, a pre‐ and post‐training assessment study was conducted in India, which evaluated the impact of training on poultry farming practices after 3 months (Alagesan et al. 2025). In Vietnam, a much longer 3‐year randomised controlled intervention study was conducted to assess the change (reduction) of AMU in small‐scale poultry farms following training (Phu et al. 2021). However, evidence on the durability of the effect of structured training programmes based on the One Health approach, emphasising poultry farm biosecurity measures in socio‐economic settings comparable to Bangladesh, remains limited.

The One Health paradigm embraces strategic and integrated risk management approaches to the safeguarding of human, animal, plant, and environmental health, and is a crucial component of sustainable agricultural development (FAO 2007). One Health frameworks aim to promote collaboration and co‐operation across health sectors, which includes the development and roll‐out of ‘One Biosecurity’ strategies to address infectious disease risks more effectively (Hulme 2020). Evaluation of training that takes a One Health approach has shown this to be effective in improving participants' understanding and implementation of biosecurity practices. For example, a study assessing the impact of training among medical, veterinary, ecology, and allied health students found that participants gained specific knowledge about biosecurity. Importantly, it found that they also applied One Health principles in other areas of their practice (Pribadi et al. 2022), suggesting that this kind of training fosters interdisciplinary collaboration and holistically addresses health risks. Therefore, this study aimed to assess whether a One Health‐based training programme can enhance biosecurity practices among poultry farmers in Bangladesh.

We designed this study to address the following research questions:

1. Does a training programme that incorporates One Health principles of learning lead to improved biosecurity practices in poultry farms?
2. How does the farm production system influence the decision‐making process related to biosecurity?
3. What impact does the farmer's level of education and farming experience have on the adoption of biosecurity measures?

In line with previous evidence, we hypothesised that production system, educational attainment, and farming experience would each influence both baseline biosecurity performance and the degree of improvement following training. Specifically, we expected that farmers operating more intensive production systems, and those with higher levels of education and longer farming experience, would demonstrate stronger adoption and retention of poultry farm biosecurity and management practices.

## 2 Materials and Methods

### 2.1 Study Sites and Period

**FIGURE 1:**
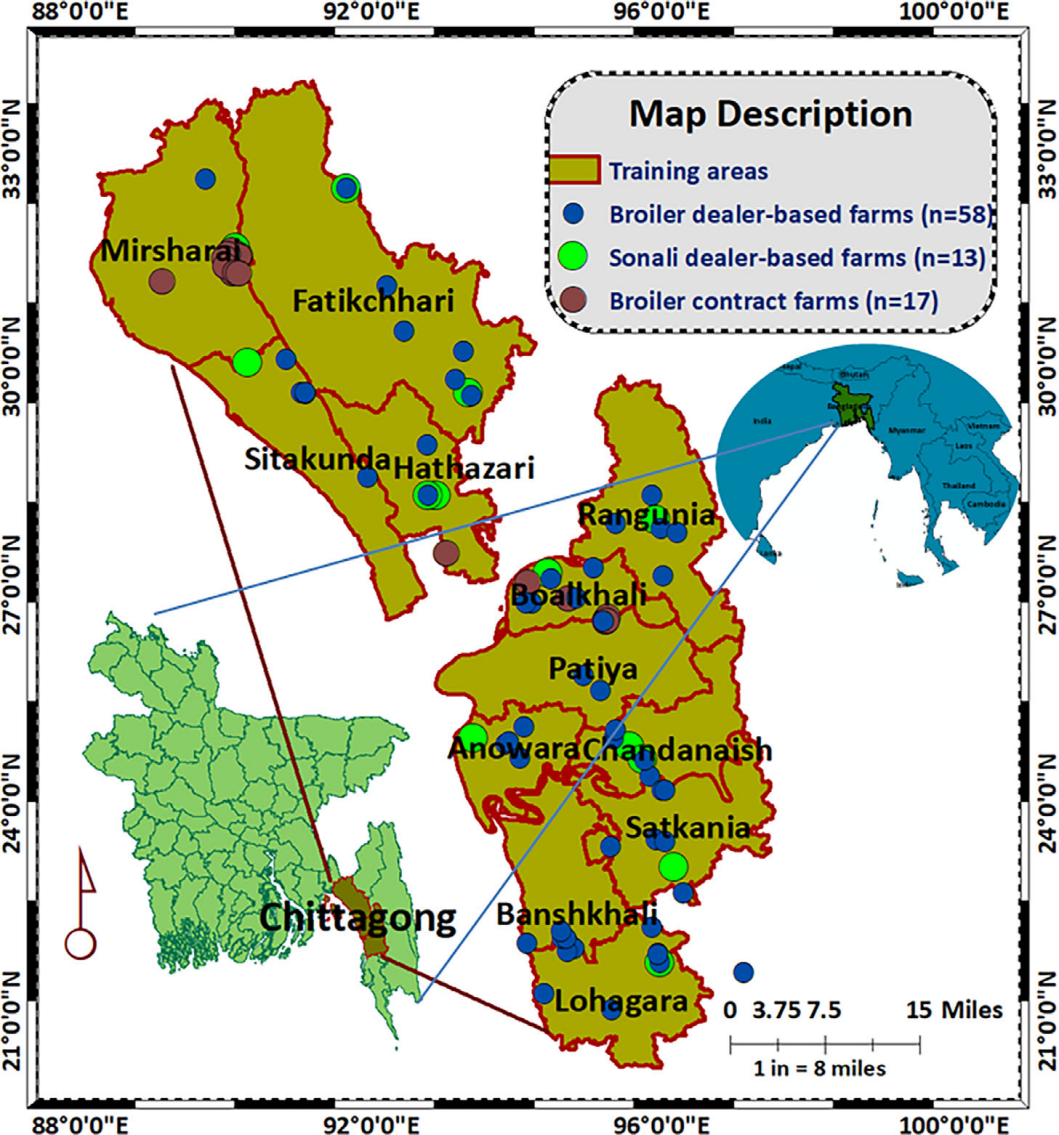
Distribution of the 88 farms in 12 upazilas of Chattogram district that were visited during the study for hands‐on training and data collection.

This pre‐ and post‐training impact assessment was conducted on 88 farms of 12 upazilas of Chattogram. Three types of farms were included (58 dealer‐based broiler [DBB] farms, 17 company‐based contract broiler [BC] farms, 13 dealer‐based Sonali [DBS]) (Table S1). In the commercial poultry sector, dealers act as intermediaries, supplying broiler and Sonali day‐old chicks (DOCs) along with other farming inputs to farmers, whereas contract farms receive broiler DOCs and feed, along with medicines and vaccines in some cases, directly from their companies under formal agreement, without intermediary involvement (Islam et al. 2023). Purposive sampling was used, in which farm locations and farmers' willingness (Figure 1) to participate were prioritised from the 12 upazilas where the One Health Poultry Hub, Bangladesh, organised training programmes with 183 small and medium‐scale commercial poultry farmers. The study period was 3 months.

### 2.2 Training Approach and Evaluation

The training approach consisted of 2 days of participatory work grounded in adult learning principles (Knowles 1984), emphasising problem‐centred and new solution development through practice and experience relevant and impactful for the farmers' activities. On Day 1, there were classroom‐based discussions regarding DOC's quality, brooding management, vaccine application, application of biosecurity measures, common poultry diseases and their preventive measures, antimicrobial usage, and management of chickens for marketing. The specific part of the training focused on the One Health principles, covering zoonotic diseases like avian influenza, salmonellosis, colibacillosis, and campylobacteriosis, as well as AMR and the environmental factors that contribute to their spread. The training's versatility came from a diverse team of veterinarians (from government, private, and academia), poultry experts, and human doctors. This was followed on the second day by practical training, which combined visits by the group of trainers and farmer trainees to seven to eight farms (belonging to the farmers who were being trained) with the first round of data collection (first assessment). At each farm, biosecurity and farm management practices were assessed using a structured questionnaire. The same farms were reassessed 4 months after the training event using the same structured questionnaire for the second round of data collection (second assessment).

### 2.3 Questionnaire and Data Collection

A structured questionnaire was developed in English and Bengali, which included questions related to farm and farmers' demographic information, including farm management and biosecurity practices (Figure S1). At the start of the training session, the questionnaire was piloted on three farms (two exotic broiler farms and one Sonali farm) to identify gaps and calibrate the timing for delivery. To assess clarity, relevance, and suitability for the local farming context, epidemiologists, and poultry experts, and field veterinarians reviewed the questionnaire, and revisions were made based on their feedback. Reliability was strengthened through piloting, interviewer training, and standardisation of question delivery to minimise interpretation and interviewer bias. The data collection was researcher‐led, and three trained veterinarians with face‐to‐face interrogation in Bengali with the participating farmers completed the questionnaire. In addition, farm records were consulted if available, and observations of the farms and ongoing practices were made.

### 2.4 Calculation of Farm Score

A poultry farm scoring sheet was prepared for individual farms following the Food and Agriculture Organization (FAO) and Department of Livestock Services (DLS) guidelines (FAO 2024; UNIDO 2023). Twenty‐seven different farm biosecurity traits were used to calculate the total score for a farm. If a trait was met positively, a full point was given; if a trait was met 50% positively, a half point was given; if a trait was met < 50% positively or non‐existent, no point was given (Table S3). All traits were equally weighted for scoring because validated, risk‐based weighing criteria of biosecurity measures for poultry farms in Bangladesh are not yet available. Equal weighting was used to provide a simple and transparent scoring system, consistent with the primary aim of the study. The list of farm traits with their description is shown in Table S2. Individual scores for each farm were calculated for both the first and second assessments.

### 2.5 Ethical Consideration

Ethical approval was obtained from Chattogram Veterinary and Animal Sciences University, Bangladesh (Ethics Application CVASU/Dir (R&E) EC/2020/165/2/1) on each occasion that the questionnaire was completed. Verbal consent was taken from the farmers before conducting the questionnaire.

### 2.6 Data Analysis

The raw data was entered using Microsoft Excel 2021, and the dataset underwent several data management procedures, including data cleaning, coding, recoding, and sorting as per the required data analysis. Cleaned data were then exported to STATA‐18 for analysis.

Univariate descriptive statistics were performed on the data of defined categories (categorical data) of variables: farm biosecurity and other practices conducted in three production systems: DBB, DBS and BC farms; between farmers' education level (Level I no formal education to primary; Level II secondary level to more) and farmers' experience in poultry farming (Experience I up to 5 years; Experience II above 5 years). The results were expressed as improvement percentages (the number of improved farms was divided by the number of farms where the farmers did not follow the measures properly before obtaining the training, among 88 farms, multiplied by 100). The formula is:

$$\frac{\mathrm{Number}\;\mathrm{of}\;\mathrm{improved}\;\mathrm{farms}\;(\mathrm{second}\;\mathrm{assessment})}{88-\mathrm{number}\mathrm{of}\mathrm{improved}\;\mathrm{farms}\;\mathrm{in}\;\mathrm{first}\;\mathrm{assessment}}\times 100$$

Paired *t*‐tests were conducted on the farm scores from the same farm at two time points (during the first and second assessments). Normality of the differences was checked using the Shapiro–Wilk test and histogram. Minor deviations were observed that the central limit theorem supports the robustness of the test for our sample size. One‐way ANOVA and independent *t*‐test for farm scores between the categories of each variable were conducted within each assessment phase according to the production system (DBB/DBS/BC), and farmers' education (Level I/Level II) and experience (Experience I/Experience II) to assess the change of farm score. The results were expressed as mean, standard error (SE) and *p* value. A correlation table between the mean scores of the first and second assessments was produced. Further regression modelling was not performed due to the small sample size and lack of heterogeneity across these categories.

## 3 Results

### 3.1 Changes in the Score of Farm Practices From the First Assessment to the Second Assessment

The farm mean score was significantly changed between the first and second assessments, irrespective of the production systems, education level, or experience level (Table 1). Interestingly, the assessment scores were not significantly different between any categories, and all farmers readily adopted some changes to practice after the training.

**TABLE 1:** Comparison of overall farm scores between two assessments (total score = 27, each question is equivalent to 1).

Categories	Variables (number of farms)	First assessment (mean ± SE)	Second assessment (mean ± SE)	*p* (paired *t*‐test)
Overall		12.3 ± 0.3	14.8 ± 0.4	< 0.0001
Production systems	Dealer‐based broiler farms (DBB) (58)	12.5 ± 0.3	14.9 ± 0.4	< 0.0001
	Dealer‐based Sonali farms (DBS) (13)	11.5 ± 0.8	14.2 ± 1.1	0.03
	Contract broiler farms (BC) (17)	12.5 ± 0.8	14.8 ± 0.9	0.01
*p* (one‐way ANOVA test)		0.3	0.8	
Education level	Level I (20)	12.1 ± 0.7	15 ± 0.9	0.001
	Level II (68)	12.4 ± 0.3	14.8 ± 0.4	< 0.0001
*p* (independent *t*‐test)		0.7	1.0	
Experience level	Experience I (46)	11.8 ± 0.4	14.7 ± 0.5	< 0.0001
	Experience II (42)	12.9 ± 0.4	14.9 ± 0.5	0.0002
*p* (independent *t*‐test)		0.6	0.9	

### 3.2 Influence of the Variables in Changing Farm Scores Between Two Assessments

**FIGURE 2:**
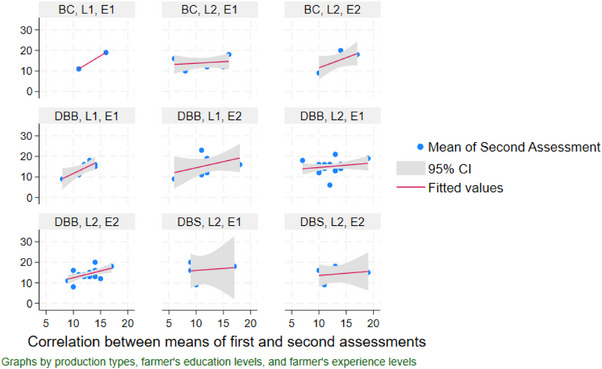
Correlation between the mean scores of the farms from the first and second assessments considering the influence of the variables: production systems, farmer's education and experience levels of the poultry farms (production systems: DBB, dealer‐based broiler farm; DBS, dealer‐based Sonali farm; BC, contract broiler farm; farmer's education level: L1, Level I, no formal education to primary; L2, Level II, secondary to more; farmer's experience level: E1, Experience I, up to 5 years; E2, Experience II, above 5 years).

The BC farms, regardless of the farmer's education and experience level, show a positive correlation (*r*: 0.44, CI: 0.27–0.61, *p* < 0.001) between two assessment scores (first and second), indicating that high first assessment scores lead to higher second assessment scores (Figure 2). However, Experience I and Level I education categories allow a more consistent positive correlation and narrower confidence intervals with different production systems than Experience II and Level II education categories. Figure 2 demonstrates various strengths of correlation across different production systems, farmers' education and experience levels through a wider confidence interval in certain panels denoting smaller sample sizes for those groups.

### 3.3 Changes in Poultry Farm Biosecurity and Other Practices Irrespective of Farm Type

**FIGURE 3:**
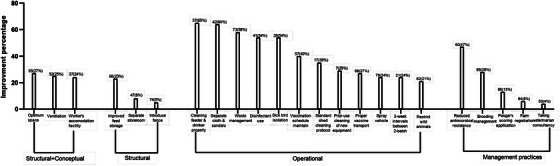
Overall (*N* = 88) improvement percentage of different biosecurity measures and management practices irrespective of farm type after at least 4 months of training organised by One Health Poultry Hub, Bangladesh.

**FIGURE 4:**
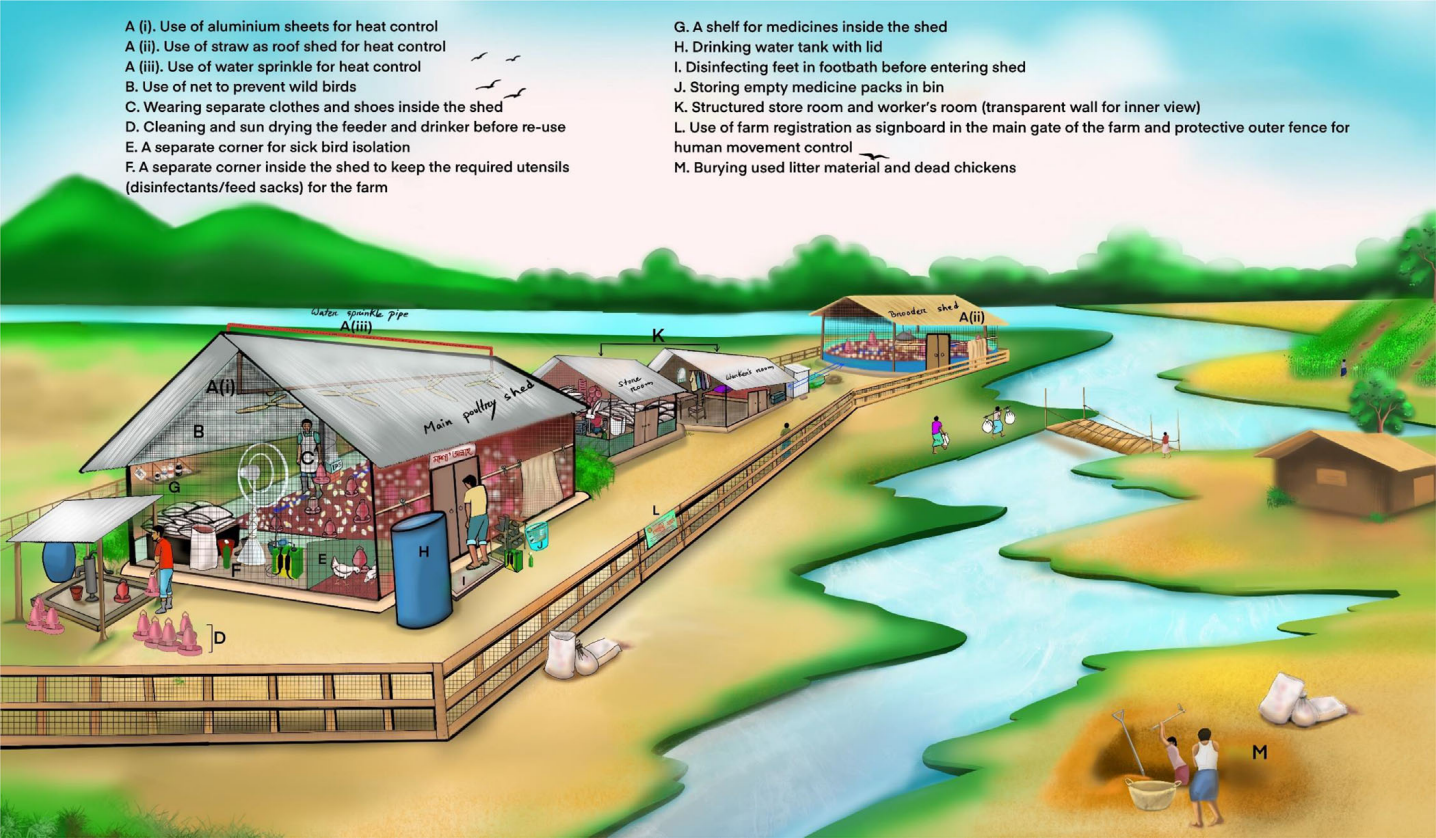
The improved poultry farming and biosecurity practices in the context of Bangladesh that help to ensure prevention of zoonotic diseases and other public health hazards like AMR, focusing on the concept of One Health.

Among the three types of biosecurity measures, the farmers improved different operational biosecurity measures mostly (Figure 3). Figure 4 is given as an illustration of various farm management and biosecurity measures in an improved way in the context of Bangladesh.

### 3.4 Changes in Poultry Farm Biosecurity and Other Practices Based on Production Systems

**FIGURE 5:**
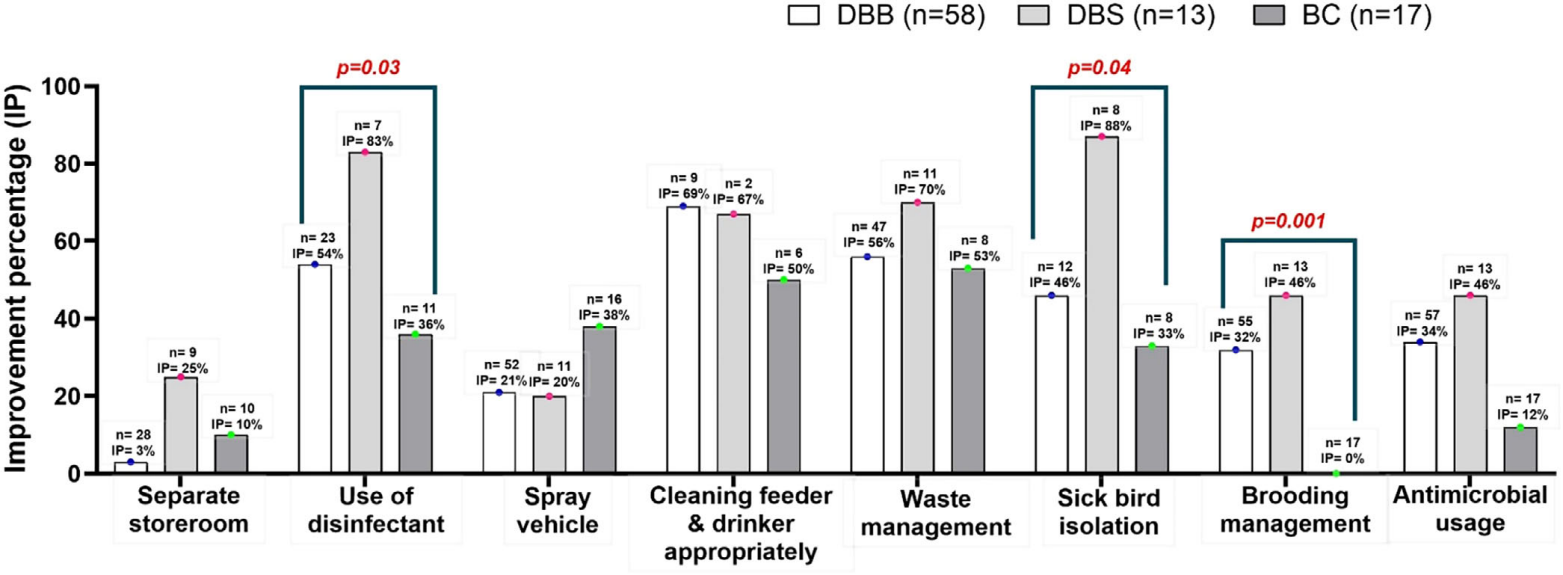
Improvement percentage of farm biosecurity measures and management practices at the farm level based on variation of production system after at least 4 months of training. BC, contracted broiler farms; DBB, dealer‐based broiler farms; DBS, dealer‐based Sonali farms; *n*, number of farms that did not practise proper farm biosecurity measures and management practices according to the first assessment.

The changes to practice of using disinfectants (83%) and sick bird isolation (88%) were significantly higher in DBS followed by DBB (disinfectant use: 54%; sick bird isolation: 46%) and BC (disinfectant use: 36%; sick bird isolation: 33%) (*p* < 0.05). The other notable change was in brooding management practices. The improvement of brooding management was 46% in DBS and 32% in DBB (*p* = 0.001) (Figure 5).

### 3.5 Changes in Poultry Farm Biosecurity and Other Practices Based on Farmers' Education and Experience Levels

**FIGURE 6:**
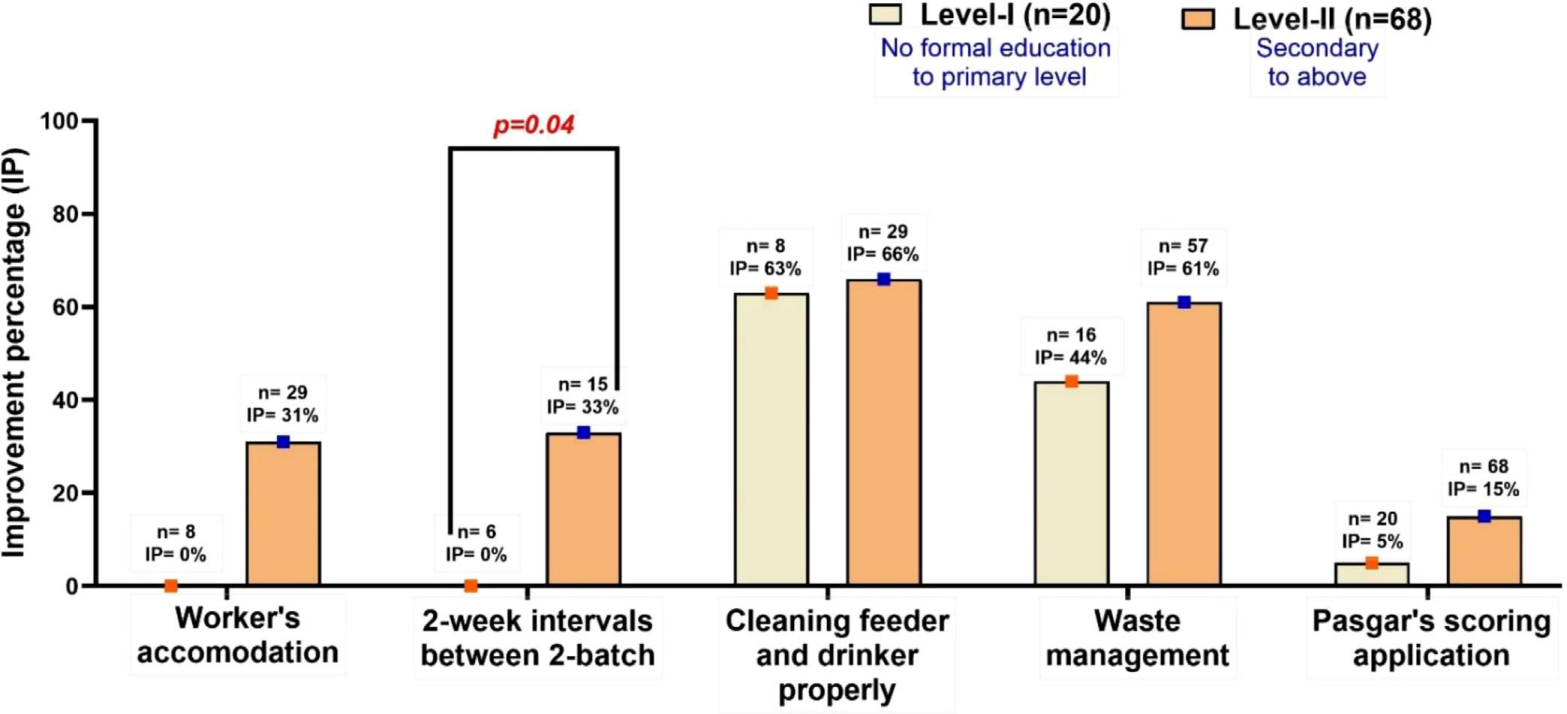
Improvement percentage of farm biosecurity measures and management practices at the farm level based on variation of farmers' education level after at least 4 months of training. Level I, farmers who had no formal education to primary education; Level II, farmers who had secondary to above education level; *n*, number of farms that did not practise proper farm biosecurity measures and management practices according to the first assessment.

Regardless of the farmers' level of education, Figure 6 shows the improvement for different types of biosecurity measures and farming practices. Farmers in the Level II education category significantly maintained the 2‐week interval between two batches (33%, *p* = 0.04), cleaning of feeders and drinkers appropriately (66%), waste management (61%), and the introduction of Pasgar's scoring application more than Level I educated farmers (Figure 6).

**FIGURE 7:**
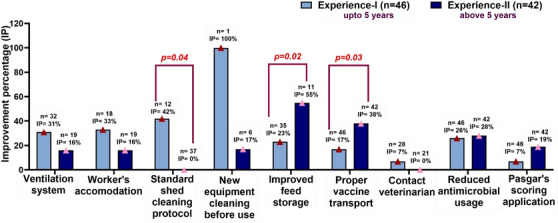
Improvement percentage of farm biosecurity measures and management practices at the farm level based on variation of farmers' experience level after at least 4 months of training. Experience I, farmers who had up to 5 years poultry farming experience; Experience II, farmers who had more than 5 years poultry farming experience; *n*, number of farms that did not practise improved farm biosecurity measures and management practices before training.

In the case farmer's experience levels, significant improvements were found in shed cleaning (Experience I: 42%, Experience II: 0%; *p* = 0.02), feed storage system (Experience II: 55%, Experience I: 23%; *p* = 0.04), proper storage of vaccine (Experience II: 38%, Experience I: 17%; *p* = 0.03) and application of Pasgar's scoring (Experience II: 19%, Experience I: 7%; *p* = 0.08) (Figure 7). The improvements of three types of biosecurity measures and farm management practices were higher in the Experience I level than in the Experience II level farms.

## 4 Discussion

This study reveals a significant improvement of poultry farming practices and biosecurity measures at varied levels across various commercial chicken farms, regardless of production system, farmers' education, and experience levels. This is a clear indication of the impact of the farmers' training. Farmers improved several recommended practices given during the training, for measures to prevent endemic and zoonotic diseases on the farm (Figure 4). Absolute improvement based on farm biosecurity scores was substantial, with mean scores increasing from 12.3 to 14.8 (out of 28) (Table 1), representing approximately 9% improvement. This indicates statistical significance and practical relevance, as these changes are expected to reduce disease transmission, improve flock health, and enhance productivity.

There was a difference in the improvements based on whether they were structural or operational biosecurity measures. Farmers increased operational biosecurity measures such as proper cleaning of feeders, drinkers, poultry sheds, and new equipment prior‐use, use of separate sandals and shoes within the farms, waste management, disinfectant use, restricting rodents and wild animals, proper vaccination schedule, and transport (Figure 3). Similar studies on the assessment of longer‐term impact have demonstrated that operational biosecurity has been improved after different periods of training intervention (Alagesan et al. 2025; Amalraj et al. 2024; Tung et al. 2020). In the present study, farm biosecurity and management practices were reassessed approximately four months after the training, using the same indicators applied during the baseline assessment. Although Swargiary (2024) reported a decline in mean knowledge retention over time, our findings demonstrated a 9% overall improvement in farm biosecurity and management practices at follow‐up. This suggests that despite the expected attenuation of knowledge, the training was associated with sustained and measurable positive changes in on‐farm practices.

Financial constraint is a factor that limits farmers' ability to maintain farm biosecurity measures (Rimi et al. 2017; Tsegaye et al. 2023; Tung et al. 2020). Changes to structural biosecurity measures require time and financial input. As the impact of the training was assessed at least four months after the training, it is possible that time and financial restrictions constrained the adoption of structural biosecurity measures. To evaluate the impact on structural biosecurity, long‐term studies are therefore necessary. In addition, qualitative studies that use focus groups and interview methods are essential to understand the reasons for not adopting biosecurity measures. Besides, subsidies or cost‐sharing or cooperative programmes and training for biosecurity implementation, locally available biosecurity measures have been shown to improve uptake in similar contexts (Islam et al. 2023).

Learning requires cognitive input with active mental engagement or processing of information using activities to promote learner engagement, understanding, organising information, and integrating it with prior knowledge. Without such input, meaningful learning is unlikely to happen. Training for adults with different levels of educational and experience requires different pedagogical approaches (Cherukunnath and Singh 2022; Hardy et al. 2015). It is important to relate to farmers' own existing experiences and knowledge. Therefore, the training programme in this study used disease‐based scenarios for discussion where the farmer can relate to their own experiences of common diseases in meat‐type poultry, including some zoonoses from poultry (Grace et al. 2024). To accommodate diverse education levels, visual aids, demonstrations, and simplified language supported lower‐literacy participants, while more experienced farmers got automatically engaged in scenario‐based problem‐solving and peer learning. This approach promoted understanding and active participation, though some participants required additional guidance, highlighting the need for ongoing reinforcement. The training emphasised FAO's (Anon 2008) core biosecurity components—segregation, cleanliness, and disinfection—as key strategies for managing farm clinical situations.

Results from the current study showed higher farm scores across all types after training, including a positive correlation of consistent improvement in DBB and BC farms with the farmers of lower educational and experience levels compared to other categories (Alagesan et al. 2025). As BC farmers have an agreement with their companies to sell the market‐ready chickens at a prior‐fixed price, they tend to decrease the production cost by monitoring the disease occurrence in the farms (Begum et al. 2013). In contrast, DBB and DBS farmers primarily operate their farms on credit provided by the dealers, which obligates them to adhere to the dealers' recommendations. These recommendations often include administering antibiotics as prophylaxis and growth promoters (Hennessey et al. 2021; Kalam et al. 2021; Masud et al. 2020). The strong influence of dealers on farmer decision‐making suggests that future interventions need to extend beyond the farm level. Engaging dealers through targeted training, certification, or stewardship initiatives may support the promotion of biosecurity practices in place of prophylactic antibiotic use. Parallel strategies to strengthen veterinary advisory services and improve farmers' independent access to reliable information could also help reduce reliance on dealer‐driven recommendations. At the same time, Sonali growers hesitate to enhance farm biosecurity because Sonali chickens have lower disease prevalence and mortality due to stronger immune systems (Hossen et al. 2021).

Rodents and wild birds can carry major zoonoses, for example, salmonellosis, campylobacteriosis and avian influenza (Grace et al. 2024). To restrict rodents and wild birds, the farmers increased farm monitoring, extra nets surrounding the shed, properly structured sheds with rodenticides and traps (Tasmim et al. 2023). A standard cleaning protocol for Bangladesh poultry sheds has been developed during the training based on expert opinions, which was suggested to the farmers to implement to prevent zoonoses and public health risks. The shed is required to be treated with disinfectant in different layers and left for 2 weeks at least, after discarding the past‐flock's used litter material and cleaning the shed properly, to remove gases from the shed. The poultry shed accumulates a number of gases (e.g., volatile organic compounds, ammonia, and hydrogen sulphide) that are produced from the poultry manure, leading to different poultry diseases and human health hazards such as conjunctivitis, irritation in the mucosa, and a runny nose (Konkol et al. 2022). Public health concerns from the training largely motivated the farmers' farming practices as they restructured the workers' accommodation facility adjacent to their poultry shed to ensure workers' and chickens' health benefits.

A significant improvement in waste management practices, including the handling of dead chickens, disposal of empty antimicrobial packets, and management of old litter materials, highlights the role of training in enhancing farmers' cognitive understanding (Alagesan et al. 2025). Effective waste management not only limits poultry disease spread but also contributes to combating AMR (Islam et al. 2022; Mutua et al. 2023; Tasmim et al. 2023).

To incorporate the ‘One Health’ into the training, the team included a human physician who discussed the impact of poultry farmers on public health, particularly in terms of optimising AMU and seeking veterinary consultation for improved poultry health care. Human physicians are highly regarded in Bangladesh, which may have contributed to the effectiveness of the training program for farmers. The results clearly show that farmers gained valuable knowledge and understanding that the optimisation of biosecurity measures and farming practices not only improves poultry health but also reduces rearing costs, leading to higher profits (Tung et al. 2020). A significant percentage of farmers reduced AMU during the brooding stage, as they tend to use antibiotics prophylactically at this early stage of poultry rearing (Phu et al. 2021). In addition, farmers improved vaccine administration by ensuring proper transport and adhering to regular schedules. They also enhanced brooding management by consulting veterinarians and using Pasgar's scoring (Sufina et al. 2018) method for DOC evaluation, which was a lesson in the topic of DOC quality identification during training.

Due to purposive sampling, this study may be subjected to selection bias, which could limit its generalisability beyond similar farming contexts. However, the detection of both improvements and some declines in practices indicates that purposive sampling did not materially affect the study's ability to capture genuine changes in biosecurity and farm management. Future research involving scaled‐up risk‐based interventions could provide deeper insight into the pathways of behavioural change and identify barriers to adopting structural biosecurity measures.

## 5 Conclusion

The One Health‐based training approach demonstrated a significant impact on operational biosecurity at least four months after the training, with a smaller effect on structural biosecurity. However, to assess the long‐term impact, further evaluations at different time points post‐training are necessary through multi‐year longitudinal follow‐up using on‐farm observations, farmer surveys, and community feedback. The extent of changes observed also depends on the type of investment, as the investment model often influences decision‐making. Dealer‐based investments, in particular, tend to limit the farmer's autonomy, with decisions being shaped more by the dealer than by the farmer. In addition, the farmer's existing knowledge and experience play a crucial role in the effectiveness of the training. Training programs should be designed to accommodate groups of adult learners with diverse educational backgrounds and experiences, and both the government and the private poultry industry, through collaboration, should adopt experiences and such a module of training programme. The pedagogical approach used in this study, which included group‐based discussions, collegial collaboration, and practical on‐farm training, likely contributed to the observed impacts. Peer influence and community norms also played a significant role in encouraging farmers to adopt new practices.

## Author Contributions

Conceptualisation and funding acquisition: Md. Ahasanul Hoque, Ayona Silva‐Fletcher, Meherjan Islam and Fiona Tomley. Material preparation and data collection: Md. Ahasanul Hoque, Meherjan Islam and Md. Ershadul Haque. Data analysis: Meherjan Islam, Ayona Silva‐Fletcher and Md. Ahasanul Hoque. Writing – first draft: Meherjan Islam, Md. Ahasanul Hoque and Ayona Silva‐Fletcher. Review and editing: Meherjan Islam, Ayona Silva‐Fletcher, Md. Ershadul Haque, Rashed Mahmud, Fiona Tomley and Md. Ahasanul Hoque.

## Funding

A grant from the UK Research and Innovation Global Challenges Research Fund One Health Poultry Hub (BB/S011269/1) funded this project.

## Conflicts of Interest

The authors declare no conflicts of interest.

## Supporting information




**Supporting File 1**: vms370843‐sup‐0001‐tableS1.docx.

**Supporting File 1**: vms370843‐sup‐0002‐tableS2.docx.

**Supporting File 1**: vms370843‐sup‐0003‐tableS3.docx.

**Supporting File 1**: vms370843‐sup‐0004‐figureS1.docx.

## Data Availability

The data supporting this study's findings are available from the corresponding author upon reasonable request.
