# Supplementary material for: Assessment of the Impact of a One Health Approach‐Based Training on Poultry Rearing and Farm Biosecurity Management in Bangladesh
Source: Vet Med Sci. 2026 Feb 7;12(2):e70843. doi: 10.1002/vms3.70843 (PMC12882552; doi:10.1002/vms3.70843)
Supplement: Supplementary file 1 — Supporting File 1: vms370843‐sup‐0001‐tableS1.docx. [file VMS3-12-e70843-s004.docx]

**Supplementary Table 1:** Distribution of the poultry farmers by upazilas (sub-district) and different production systems, farmer’s education levels, and farmer’s experience levels

| **Upazila**  **(n= farm number)** | **Production system** | | | **Farmer’s education levels** | | **Farmer’s experience levels** | |
| --- | --- | --- | --- | --- | --- | --- | --- |
|  | Dealer-based broiler farms (n) | Contract broiler farms (n) | Dealer-based Sonali farms (n) | Level-I  (no formal education to primary) (n) | Level-II  (Secondary to more) (n) | Level-I  (up to 5 years) (n) | Level-2  (above 5 years) (n) |
| Anowara (9) | 6 |  | 3 | 3 | 6 | 5 | 4 |
| Banshkhali (8) | 8 |  |  | 5 | 3 | 4 | 4 |
| Boalkhali (12) | 5 | 6 | 1 | 0 | 12 | 8 | 4 |
| Chandanaish (9) | 6 |  | 3 | 4 | 5 | 4 | 5 |
| Fatikchhari (8) | 6 |  | 2 | 1 | 7 | 3 | 5 |
| Hathazari (5) | 3 |  | 2 | 0 | 5 | 3 | 2 |
| Lohagara (7) | 6 |  | 1 | 2 | 5 | 6 | 1 |
| Mirsharai (7) |  | 10 |  | 3 | 7 | 5 | 5 |
| Patiya (2) | 2 |  |  | 0 | 2 | 0 | 2 |
| Rangunia (7) | 6 |  | 1 | 1 | 6 | 3 | 4 |
| Satkania (4) | 4 |  |  | 1 | 3 | 3 | 1 |
| Sitakunda (7) | 5 | 1 | 1 | 0 | 7 | 2 | 5 |
| **Total (88)** | **58** | **17** | **13** | **20** | **68** | **46** | **42** |
