# Supplementary material for: Assessment of the Impact of a One Health Approach‐Based Training on Poultry Rearing and Farm Biosecurity Management in Bangladesh
Source: Vet Med Sci. 2026 Feb 7;12(2):e70843. doi: 10.1002/vms3.70843 (PMC12882552; doi:10.1002/vms3.70843)
Supplement: Supplementary file 2 — Supporting File 1: vms370843‐sup‐0002‐tableS2.docx. [file VMS3-12-e70843-s001.docx]

**Supplementary Table 2:** The list of the farm traits considered for scoring the visited farms in the impact assessment (during first and second assessment) study of the training programme of One Health Poultry Hub, Bangladesh

| **Serial No** | **Farm traits** |
| --- | --- |
| 1. M | **Farm registration (Yes/No)** |
| 2. S+C | **Ventilation system:** open shed, provide electric fans with dynamos when electricity fails, water sprinkle, and use of asbestos/aluminum sheets, jute sacks to improve and manage temperature control within the shed. |
| 3. C+O | **Space:** give one sq/ft area to two chicks up to two weeks of age and one sq/ft area to one adult  chicken |
| 4. S | **Fence** around farm (Yes/No) |
| 5. S+O | **Restrictive measures against wild and domestic animals (e.g., domestic chickens and ducks, stray dogs and cats, foxes, civets, wild birds):** use of rodenticides and rat traps, extend the shed-  roof, block the entrance of wild birds, use the net to prevent the birds and rodents, and cut the trees |
| 6. S | **No nearby farm/no access other species (e.g., pigeon, Fayoumi, indigenous chickens and ducks, quail, cattle, goats) to farm** |
| 7. S | **Separate storeroom for equipment and feed storage** |
| 8. S+C | **Staff accommodation inside the shed** (ensure workers safety with a separate room near the poultry shed so that they can monitor the farms on time and at regular interval, including subsistence and  separate washroom facilities) |
| 9. S | Any **live bird market (LBM)** within **1 km** |
| 10. S | The **main road** within **500 meters** |
| 11. O | **Use of disinfectants** (e.g., soap, savlon, detergent, lime, potash, phenol, available market products,  for example, Timsen, Virocid, GPC-8) as the spray or in the footbath |
| 12. O | **Use separate sandals and clothes** while working in the farm |
| 13. O | Allow the **vehicles** inside the shed premises, or spray the vehicles before entering the shed |
| 14. O | **Follow the all-in-all-out method** |
| 15. O | **Follow the 14-day gap between two successive batches** |
| 16. O | **Follow standard shed cleaning measures:** if the farm floor is mud- cleaned by removing the litter material properly, keeping the sheds empty for a few days to ensure any residual ammonia gas expulsion) re-surfacing the floor with mud and paint, repairing the holes of the shed and use disinfectant on the floor and surroundings of the shed. In the case of a paved-floor farm, the floor  is cleaned using water and detergent or soap and is kept unused until free of ammonia gas smell. |
| 17. O | **Follow the appropriate feeder and drinker cleaning method:** washing the drinkers daily with water, scrub, and soap (once/twice/thrice) and the feeders after the batch-end with soap and water, also mopping the feeder with disinfectants sometimes and washing the feeders with water and soap  or disinfectant if having two sets of feeders. |
| 18. O | **Clean new equipment before introducing to the shed** |
| 19. O | **Feed storage management** (keep dry and free from vermin) |
| 20. O | **Waste disposing system:** selling or using litter as fertilizer in the cultivation land, storage of empty medicine packets to burn or sell, and burying the dead birds or discarding them in a pit for bio-gas  production. |
| 21. O | **Sick bird isolation** |
| 22. O | **Vaccine transport and schedule** |
| 23. O | **Distance between two sheds** (50 feet between two sheds) |
| 24. M | **Communicate with the veterinarians** |
| 25. M+C+O | **Brooding management** |
| 26. M | **Concern about antimicrobial resistance:** reducing the use of antibiotics, increasing the use of  probiotics, maintaining the withdrawal period, not selling the chickens during the course of antibiotics |
| 27. M | **Apply the Pasgar’s scoring technique** to assess bird quality |

[S= Structural biosecurity measure, C= Conceptual biosecurity measure, O= Operational biosecurity measure, M= management practices]
