# Supplementary material for: Assessment of the Impact of a One Health Approach‐Based Training on Poultry Rearing and Farm Biosecurity Management in Bangladesh
Source: Vet Med Sci. 2026 Feb 7;12(2):e70843. doi: 10.1002/vms3.70843 (PMC12882552; doi:10.1002/vms3.70843)
Supplement: Supplementary file 3 — Supporting File 1: vms370843‐sup‐0003‐tableS3.docx. [file VMS3-12-e70843-s002.docx]

**Supplementary table 3:** Rubric table for scoring the list of the farm traits while visiting farms in the impact assessment (during first and second assessment) study of the training programme of One Health Poultry Hub, Bangladesh

| **Farm traits** | **Considering characteristics for**  **100% score (Point 1)** | **Considering a 50% score (Point 0.5)** | **No point (Point 0)** |
| --- | --- | --- | --- |
| Farm registration | Yes | - | No |
| Ventilation system | Open shed, provide electric fans with dynamos when electricity fails, water sprinkle, and use of asbestos/aluminum sheets, jute sacks to improve and manage temperature control within the shed. | Open shed with the use of jute sacks to improve and manage temperature control within the shed | Closed and stagnant shed. No air circulation. |
| Space | give one sq/ft area to two chicks up to two weeks of age and one sq/ft area to one adult chicken | - | No optimum space |
| Fence around farm | Yes | - | No |
| Restrictive measures against wild and domestic animals (e.g., domestic chickens and ducks, stray dogs and cats, foxes, civets, wild birds) | Restrictive measures against wild and domestic animals: Use of rodenticides and rat traps, extend the shed-roof, block the entrance of wild birds, use the net to prevent the birds and rodents, and cut the trees | Restrictive measures against wild and domestic animals: Any of these:  1. Use of rodenticides and rat traps  2. extend the shed-roof  3. block the entrance of wild birds  4. use the net to prevent the birds and rodents  5. cut the trees | No restrictive measures |
| No nearby farm/no access other species (e.g., pigeon, Fayoumi, indigenous chickens and ducks, quail, cattle, goats) to farm | No nearby farms | - | Present of neighbouring farms |
| Staff accommodation inside the shed | Ensure workers safety with a separate room near the poultry shed so that they can monitor the farms on time and at regular interval, including subsistence and separate washroom facilities | Almost a good separate room for the workers | No |
| Any live bird market (LBM) within 1 km | No | - | Yes |
| The main road within 500 meters | No | - | Yes |
| Use of disinfectants (e.g., soap, savlon, detergent, lime, potash, phenol, available market products, for example, Timsen, Virocid, GPC-8) | Use of disinfectants as the spray or in the footbath | Partial use of disinfectants | No |
| Use separate sandals and clothes while working in the farm | Strict use of separate sandals and clothes inside the shed | Partial use of separate sandals and clothes | No use |
| Allow the vehicles inside the shed premises, or spray the vehicles before entering the shed | Do not allow vehicles and also use disinfectants as spray | Allow vehicles but spray disinfectants | Allow vehicles inside the farm premise without spray |
| Follow the all-in-all-out method | Yes | - | No |
| Follow the 14-day gap between two successive batches | Yes | - | No |
| Follow standard shed cleaning measures | If the farm floor is mud- cleaned by removing the litter material properly, keeping the sheds empty for a few days to ensure any residual ammonia gas expulsion) re-surfacing the floor with mud and paint, repairing the holes of the shed and use disinfectant on the floor and surroundings of the shed. In the case of a paved-floor farm, the floor is cleaned using water and detergent or soap and is kept unused until free of ammonia gas smell. | Do not use disinfectants properly to clean the shed | Irregularity in shed cleaning measures |
| Follow the appropriate feeder and drinker cleaning method | Washing the drinkers daily with water, scrub, and soap (once/twice/thrice) and the feeders after the batch-end with soap and water, also mopping the feeder with disinfectants sometimes and washing the feeders with water and soap or disinfectant if having two sets of feeders. | Do not use disinfectants properly to wash the drinkers well | Irregularity in cleaning feeder and drinker |
| Clean new equipment before introducing to the shed | Yes | - | No |
| Feed storage management | Keep on high table to keep it dry and protect from the rodents | Protect from the rodents | No measure to keep the feed well |
| Waste disposing system | Selling or using litter as fertilizer in the cultivation land, storage of empty medicine packets to burn or sell, and burying the dead birds or discarding them in a pit for bio-gas production | Maintain only one good measure to dispose of the waster | No measure |
| Sick bird isolation | Yes | - | No |
| Vaccine transport and schedule | Maintain properly | Irregularity to maintain these measures properly | Do not measure |
| Distance between two sheds (50 feet between two sheds) | Yes | - | No |
| Communicate with the veterinarians | Yes | - | No |
| Brooding management | Maintain proper brooding temperature, feeding and care management of the chicks | Maintain partially | No measure |
| Concern about antimicrobial resistance | Reducing the use of antibiotics, increasing the use of probiotics, maintaining the withdrawal period, not selling the chickens during the course of antibiotics | Maintain any single measure | Not concern |
| Apply the Pasgar’s scoring technique to assess bird quality | Yes | - | No |
