# Supplementary material for: Assessment of the Impact of a One Health Approach‐Based Training on Poultry Rearing and Farm Biosecurity Management in Bangladesh
Source: Vet Med Sci. 2026 Feb 7;12(2):e70843. doi: 10.1002/vms3.70843 (PMC12882552; doi:10.1002/vms3.70843)
Supplement: Supplementary file 4 — Supporting File 1: vms370843‐sup‐0004‐figureS1.docx. [file VMS3-12-e70843-s003.docx]

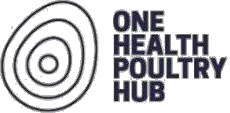

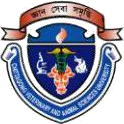

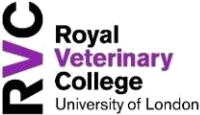


The Evaluation copy of existing biosecurity measures and activities of small and medium scale broiler and Sonali farm of Chattogram, Bangladesh

| 1. Name of the farmer: |  | | | |
| --- | --- | --- | --- | --- |
| 2. Type of the farm: | a) Broiler | b) Sonali |  |  |
| 3. Investment type of farm: | a) Credit | b) Half-credit | c)Cash | d) Contracted |
| 4. Farm registration: | 1. Yes, Date of registration: 2. Not registered | | | |
| 5. Address: |  | | | |
| 6. Main source of income: |  | | | |
| 7. Experience of farming: | 9. Age of the chickens: | | | |
| 8. Mobile number: | 10: Area of the shed: | | | |
|  | 11. Number of chickens: | | | |


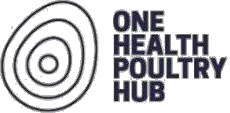

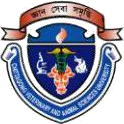

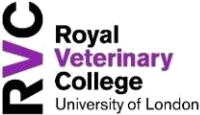


| **SL**  **No** | **Biosecurity measures** | **Pre-findings** | | | | **Post-findings** | | | |
| --- | --- | --- | --- | --- | --- | --- | --- | --- | --- |
|  |  | **Yes** | **No** | **Comment** | **Number**  **(28)** | **Yes** | **No** | **Comment** | **Number**  **(28)** |
| 1. | Is there available ventilation in the shed (sufficient fans)? Do they use jute sack as the  curtain? |  |  |  |  |  |  |  |  |
| 2. | Is there sufficient accommodation of the chicken inside the shed? (2sqft for two chicks up to 2 weeks and 1sqft for an adult  chicken up to sale) |  |  |  |  |  |  |  |  |
| 3. | Is there a fence surround the  shed? |  |  |  |  |  |  |  |  |
| 4. | Is there any hole in the wall/net/fence of the shed by which the wild birds, indigenous chickens or  rodents can enter? |  |  |  |  |  |  |  |  |
| 5. | Is there any measure to prevent the restricted animals inside the shed? E.g.,  rat trap |  |  |  |  |  |  |  |  |
| 6. | Is any other species of poultry (e.g., indigenous chickens, duck, pigeon, guinea fowl, quail,etc.)  reared nearby the shed? |  |  |  |  |  |  |  |  |
| 7. | Is the storeroom (for equipment and feed)  separate from farm? |  |  |  |  |  |  |  |  |
| 8. | Do the workers stay in the  farm or farm premise? |  |  |  |  |  |  |  |  |
| 9. | Is there any market nearby  the farm? (within 1km) |  |  |  |  |  |  |  |  |
| 10. | Is the farm nearby the main  road? (with 0.5km) |  |  |  |  |  |  |  |  |
| 11. | Is there footbath/ disinfectant spray in the  farm? |  |  |  |  |  |  |  |  |
| 12. | If there is a footbath, does the farmer change the water of the footbath at 24hours  interval? |  |  |  |  |  |  |  |  |


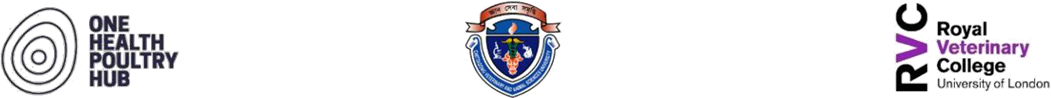


| **SL**  **No** | **Biosecurity measures** | **Pre-findings** | | | | **Post-findings** | | | |
| --- | --- | --- | --- | --- | --- | --- | --- | --- | --- |
|  |  | **Yes** | **No** | **Comment** | **Number**  **(28)** | **Yes** | **No** | **Comment** | **Number**  **(28)** |
| 13. | Do the workers/ visitors change their shoes before  entering shed? |  |  |  |  |  |  |  |  |
| 14. | Do they disinfect and check the vehicle before entering  the shed? |  |  |  |  |  |  |  |  |
| 15. | Do they maintain "All-in-All-  out" process? |  |  |  |  |  |  |  |  |
| 16. | Do the farmer keep 14days interval before two  batches? |  |  |  |  |  |  |  |  |
| 17. | If yes, is there any facility to keep the shed clean and  disinfected? |  |  |  |  |  |  |  |  |
|  | 17.1 Do they clean the feeder and drinker every  day? |  |  |  |  |  |  |  |  |
| 18. | Do they clean the new equipment before using in  the farm? |  |  |  |  |  |  |  |  |
| 19. | Do they keep the feed in a  clean and dry place? |  |  |  |  |  |  |  |  |
| 20. | Does the farmer discard the used litter material, empty packet of medicine, and dead birds in closed  dustbin? |  |  |  |  |  |  |  |  |
|  | 20.1 If no, where does he  discard the wastage? |  |  |  |  |  |  |  |  |
| 21. | How do they discard the dead birds? E.g., pit or  compost |  |  |  |  |  |  |  |  |
| 22. | Is there isolation facility in the shed for the sick  chickens? |  |  |  |  |  |  |  |  |
| 23. | Does the farmer administer vaccine regularly? E.g.,  IBD/ND vaccine |  |  |  |  |  |  |  |  |


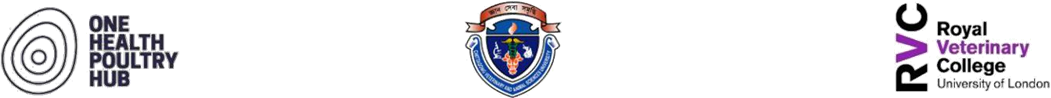


| **SL**  **No** | **Biosecurity measures** | **Pre-findings** | | | | **Post-findings** | | | |
| --- | --- | --- | --- | --- | --- | --- | --- | --- | --- |
|  |  | **Yes** | **No** | **Comment** | **Number**  **(28)** | **Yes** | **No** | **Comment** | **Number**  **(28)** |
|  | 23.1 If yes, when did he administer the last vaccine and what is the interval of  vaccination? |  |  |  |  |  |  |  |  |
| 24. | If there are many sheds in a farm, is there any distance  between the sheds? (Measure the distance) |  |  |  |  |  |  |  |  |
| 25. | Do you see the empty medicine packets in the farm premise? Many packets (more than 20)/some packets/less packets (less  than 10) |  |  |  |  |  |  |  |  |
| 26. | How is the disposal system of the farm? Very  good/Good/Bad/ Very bad |  |  |  |  |  |  |  |  |
| 27. | Do your works have any training on farm’s biosecurity  measures? |  |  |  |  |  |  |  |  |
|  | 27.1 If yes, how many times and how many farmers got  this training? |  |  |  |  |  |  |  |  |
| 28. | What diseases are mostly occurred in your farm? Do you diagnose the disease by a veterinarian or in any hospital?  IBD/ND/Colibacillosis/ Salmonellosis/ Coccidiosis/Brooder pneumonia |  |  |  |  |  |  |  |  |
|  | **Total** |  |  |  |  |  |  |  |  |


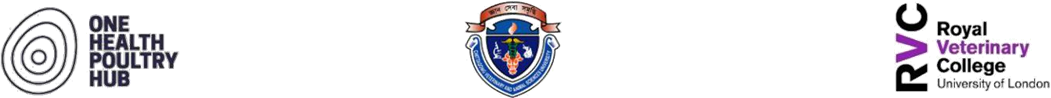


**Comment please, if you have any**

**Farm Biosecurity Evaluation Result**

| **Good sides of the farm** | **Bad sides of the farm** | **Recommendations for farm improvement** | **If there is any improvement** | |
| --- | --- | --- | --- | --- |
|  |  |  | **Yes** | **No** |
|  |  |  |  |  |
|  |  |  |  |  |
|  |  |  |  |  |
|  |  |  |  |  |
|  |  |  |  |  |
